# Annotations

**Published:** 1889-11-23

**Authors:** 


### Annotations.

**Fogs and Philosophers.** "We consume our own smoke," says Mr. John Stewart, proprietor of the Blackwall Iron Works, "and we save about 30 per cent. in the process." And yet, on this fourteenth day of November, in the year of grace eighteen hundred and eighty-nine, London is enveloped in an atmosphere of smoke and fog the like of which Dante never dreamed of finding in the worst regions of the Inferno. The November, December, January, and February fogs which, with their admixture of concentrated smoke, make our fine city the most detestable winter residence in the whole world, cost us about 30 per cent. more than we should be required to spend if we were compelled to keep our atmosphere pure. Talk of Don Quixote and his mad freaks in tilting at windmills and flocks of sheep! The Don was a perfect philosopher compared with us moderns who live in London. Royal Societies, Colleges of Physicians, Local Government Boards, County Councils—of what earthly use are they all unless they can keep us from being choked and strangled in broad day by the terrible incubus of tons of sulphurous soot? Is this mere railing? By no means! The time will certainly come when the inhabitants of this and all other large towns will be compelled to save their pockets by consuming their smoke. And when that time does come the people will assuredly not believe that their predecessors for generation after generation were so mad as to live in an atmosphere which hid both cloud and sunshine alike, kept them in darkness at midday, and killed them by inches by offering them carbon and watery vapour instead of air to breathe. The County Council has done some good work, and it has more to do. But is there anything of greater practical urgency than this question of fog and smoke? London and its suburbs for ten or fifteen miles round are not fit to be lived in in winter. Outside that radius there are clear skies and health-giving breezes. Shall we conquer the smoke enemy, or shall we be conquered by it? One or the other alternative must be our choice. If we could but get some of our philosophers who propound theories for the twenty-fifth century to work out—if we could but get them to live and work for the actual present, what a blessing it would be! In the meantime we waste our money on superfluous coal in order that we may fee the doctor and employ the undertaker, and be miserable whilst the killing is in progress. "O tempora!"

**A Rare Gift.** All men and women who understand and value the public health will recognise in Sir Sydney Waterlow a true and patriotic friend of his country. It seems somehow to be taken for granted that a possessor of land in London, however rich he may be, is entitled to ask and receive the most exorbitant price for his land, whatever be the philanthropy or public usefulness of the purpose for which it is required. Some years ago the writer was one of a committee who were seeking ground on which to erect a new hospital. There were several suitable plots in the district, the hospital was imperatively needed, and it was thought by the less sophisticated members of the committee that the proprietors of the ground would be quite willing to sell at the market price, or even a little lower, because of the undoubted value of the institution to be built. But what were the actual facts? That every proprietor who was approached, the moment he knew the ground was wanted for a hospital and not for speculative building purposes, at once raised his terms enormously. In some cases the amount asked was fully twice as much as the market value of the ground. It was not that there was any objection to the hospital, for the neighbourhood was a poor one; but that the necessity for it being great, the proprietor thought there was a fine opportunity of making a first-rate bargain. "Very natural," says the cynic and the hard man of business. "Utterly disgraceful and shameless," says the man who has humanity enough about him to wish to do as he would be done by. He who takes advantage of other men's necessities to exact more than the value of anything he has to sell, is very near the borderland of scoundrelism; whilst he who exacts more than is fair from the necessities of the sick poor is a scoundrel, and would be a thief if he dared. We write this in cold blood for the special benefit of those who think that any means of getting money are justifiable so long as they do not bring the money-getter within the clutches of the law. The public in estimating Sir Sydney Waterlow's gift of nearly thirty acres of beautifully timbered and highly valuable building ground, will do well to look at it from this point of view. What would an ordinary proprietor have said if he had been asked to sell this very ground for a public park? If its building value be £50,000 he would probably have demanded £100,000 for it; and there he would have stuck until he got it, the shameless miser! What has Sir Sydney Waterlow done? He has given the ground for nothing and £6,000 with it. It was a noble thought, Sir Sydney, and nobly carried out into act and deed! Posterity will say of you that you deserved well of your city and your country.

**Church Parades.** We cannot express much sympathy with the organisers of church parades and other Sunday demonstrations, though the avowed object is to benefit charities. One of the great charms of London, to Londoners, on Sunday is the quiet—*i.e.*, the deserted aspect of the streets. The invasion of an army of banner-bearers, men, women, and children emblazoned with emblems, and a host of enthusiastic persons armed with collecting-boxes, is not one to be welcomed by the peaceful at any rate. Still, there is something to be said on the other side. These church parades excite a warm interest among certain members of the working classes who are unable to give much time to charitable works on other days. It is further in their favour that they show steady progress in a right direction—namely, the increased amounts they are able to collect. On a recent occasion, at a church parade in support of the Victoria Hospital for Sick Children, which perambulated Chelsea, £300 was raised, of which upwards of £270 was paid to the credit of the charity. No doubt the object and the locality selected for this particular church parade had much to do with the largeness of the amount. Still, we have also evidence that these parades, even in working-class neighbourhood, are producing larger and larger sums as the people get used to them and come to understand the really excellent motives animating the promoters, whatever one may think of their methods and procedure.

**Mild, but not Safe.** A little "reminder" at the turn of the seasons is very necessary for some persons and useful for all. The weather during the last few weeks has been abnormally mild. The writer saw at Brighton, last Sunday, oxeyes, pansies, beautiful crimson roses, and several other summer flowers blooming in the open air. He heard the lark singing, too, on the downs above the town as if it had been spring. But it is not spring, nor yet is it autumn; and it is rather a dangerous time. Some people are always exercised about the kind of clothing they should wear between the seasons. A witty fellow once said, "When it is fair carry an umbrella; when it rains do as you please." So we would say, "When the season is changing from autumn to winter wear plenty of warm woollen clothing; when winter is once established, you may do as you please." For when the cold has fairly set in, you are certain to do that which makes both for comfort and health. The "reminder" is not quite exhausted when the clothing has been dealt with. Many people take daily walks in the open air, and when they are tired and hot they find a seat both "grateful and comforting," like a certain well-known cocoa. But sitting down is a thing to be done with great discretion on a mild November day. The mildness makes the walker warm, but the air, though not of a low temperature, is very moist, and therefore very chilly. He who sits down on a seat in November should make sure that the seat is dry, should keep his coat buttoned, and his hat upon his head, and should not sit more than two or three minutes at the longest. He will soon begin to feel chilly, and perhaps to sneeze. When that stage is reached he should rise and walk forward in a moment. Of course no sane person will ever think of sitting down upon the ground in November or any other autumn or winter month. The best course, indeed, is to walk very gently so as to avoid perspiring, and not to sit down at all. Innumerable colds, and not a few attacks of bronchitis and inflammation of the lungs, are got by nothing else but sitting down in the open air because the weather is so mild. Grown-up people should know how to take care of themselves, but nurses with children must be instructed not to allow them to stand still, to sit down, or to take off either hat or jacket in the open air until the winter is past and the spring of next year is well advanced.

